# Anti-Inflammatory Effects of Phytochemical Components of *Clinacanthus nutans*

**DOI:** 10.3390/molecules27113607

**Published:** 2022-06-04

**Authors:** Wei-Yi Ong, Deron R. Herr, Grace Y. Sun, Teng-Nan Lin

**Affiliations:** 1 Department of Anatomy and Neurobiology Research Programme, National University of Singapore, Singapore 119260, Singapore; 2 Department of Pharmacology, National University of Singapore, Singapore 119260, Singapore; dherr@expressiondrugdesigns.com; 3 Department of Biochemistry, University of Missouri, Columbia, MO 65211, USA; sung@missouri.edu; 4 Institute of Biomedical Sciences, Academia Sinica, Taipei 11529, Taiwan; bmltn@ibms.sinica.edu.tw

**Keywords:** *Clinacanthus nutans*, inflammation, vitexin, isovitexin, orientin, isoorientin, schaftoside

## Abstract

Recent studies on the ethnomedicinal use of *Clinacanthus nutans* suggest promising anti-inflammatory, anti-tumorigenic, and antiviral properties for this plant. Extraction of the leaves with polar and nonpolar solvents has yielded many C-glycosyl flavones, including schaftoside, isoorientin, orientin, isovitexin, and vitexin. Aside from studies with different extracts, there is increasing interest to understand the properties of these components, especially regarding their ability to exert anti-inflammatory effects on cells and tissues. A major focus for this review is to obtain information on the effects of *C. nutans* extracts and its phytochemical components on inflammatory signaling pathways in the peripheral and central nervous system. Particular emphasis is placed on their role to target the Toll-like receptor 4 (TLR4)-NF-kB pathway and pro-inflammatory cytokines, the antioxidant defense pathway involving nuclear factor erythroid-2-related factor 2 (NRF2) and heme oxygenase 1 (HO-1); and the phospholipase A_2_ (PLA_2_) pathway linking to cyclooxygenase-2 (COX-2) and production of eicosanoids. The ability to provide a better understanding of the molecular targets and mechanism of action of *C. nutans* extracts and their phytochemical components should encourage future studies to develop new therapeutic strategies for better use of this herb to combat inflammatory diseases.

## 1. Introduction

**Figure 1:**
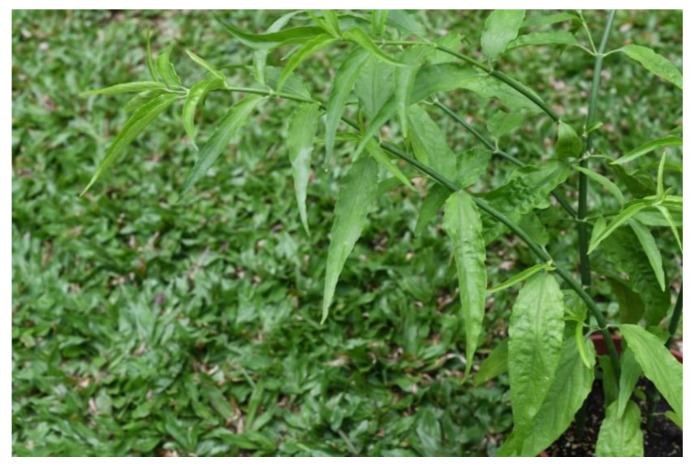
*Clinacanthus nutans* plant (Photo by W.Y. Ong).

*Clinacanthus nutans* Lindau is a perennial shrub in the Acanthaceae family that is also known by its common names belalai gajah (Malay), phaya yo (Thai), Sabah snake grass, ki tajam (Sunda), dandang gendis (Jawa), and you dun cao (Mandarin). This plant is found largely in China, Vietnam, Thailand, Malaysia, and Indonesia, and has upright, drooping branches that grow to 1–3 m tall. Its name is derived from the Greek “klinh” meaning prostrate, and “Acanthus”, referring to Acantha, a nymph loved by the Greek god Apollo who was changed into an Acanthus plant. The Latin “nutans” means nodding, referring to the drooping form of the branches and leaves. The leaves of this plant are simple, opposite, stalked, and are linear-lanceolate in shape, 4–12 cm long by 1–4 cm wide [1] (Figure 1).

*Clinacanthus nutans* has diverse uses as a traditional herbal medicine for treating skin rashes, insect and snake bites, lesions caused by herpes simplex virus, diabetes, and gout in Malaysia, Indonesia, Thailand, Singapore, and China [2,3]. In addition to its antioxidant and anti-cancer effects, a recent review of the pharmacological properties of *C. nutans* leaves also highlighted potential anti-hyperlipidemia, vasorelaxation, and renoprotective activities [4]. Early harvesting of *C. nutans* (6 months old) yielded the highest concentrations of secondary metabolites, which are potent antioxidant compounds. A key enzyme for flavonoid synthesis, chalcone synthase, is present in *C. nutans* leaves and buds, and its activity is correlated with the production of phenolic acids, which increases from 1 to 6 months, but decreases from 6 months to 1 year [5]. The antioxidant activity and phytochemical contents of *C. nutans* gradually increase with the age of the plant and peak at week 16. The content of bioactive compounds of *C. nutans* is significantly influenced by harvesting frequencies, especially regarding levels of schaftoside, isoorientin, and orientin. Among these constituents, schaftoside is the major compound at different harvesting ages and frequencies [6]. A study of crude methanol extract of *C. nutans* leaves from 11 different locations with diverse environmental conditions in Malaysia, Thailand, and Vietnam, showed that extracts from plants grown at higher elevations and lower air temperatures were more cytotoxic to D24 melanoma cells, than those grown at lower elevations and higher air temperatures [7].

The median lethal dose (LD50) of aqueous extract of *C. nutans* leaves was reported to be >5000 mg/kg, and in one study, no adverse effect was observed in rats after oral administration of 2000 mg/kg/day for 90 days [8]. In another study, a single dose of 5000 mg/kg of *C. nutans* water extract was administered to Sprague Dawley rats, and no sign of overt toxicity was found on routine toxicity evaluation for 14 days [9]. Serum ¹H-NMR metabolomic analyses also revealed no significant metabolic difference between untreated and treated groups. Urinary ¹H-NMR analysis however, showed alterations in carbohydrate metabolism, energy metabolism, and amino acid metabolism 2 h after extract administration, but these changes reverted to normal at 24 h post-administration [9]. Long term oral administration of *C. nutans* ethanolic leaf extract of 1000 mg/kg for 28 days in female ICR mice resulted in significant increases in creatinine, alanine transaminase, and moderate hepatic and renal necrosis [10]. Together, these results suggest that although a single dose of *C. nutans* extract may not have lasting cytotoxic effects, repeated oral administration of high doses of extracts (1 g/kg for 1 month) could potentially lead to toxic effects in the liver and kidneys.

In our previous work on *C. nutans*, we noticed significant effect of its ethanolic and methanolic extracts in inhibiting cytosolic phospholipase A2 (cPLA_2_), which is a key enzyme for producing arachidonic acid (ARA) and pro-inflammatory eicosanoids. The aim of this paper is the review of existing literature, not only about the anti-inflammatory effects of *C. nutans*, but also its prominent constituent phytochemicals in relation to the anti-inflammatory and antioxidant signaling pathways involving NF-kB, Nrf2, and PLA_2_, with the hope to seek a better mechanistic understanding of the herb on disease processes.

## 2. Effects of Different Extraction Methods on Biological Properties

### 2.1. Aqueous, Polar, and Semi-Polar Extractions

Recent reviews of the ethnomedicinal use of *C. nutans* suggest that the plant has promising anti-inflammatory, anti-tumorigenic, and antiviral properties. Different extraction methods have been shown to yield different compounds with anti-inflammatory, antioxidant, anti-diabetic, and antiviral effects (Table 1). Several classes of phytochemicals such as flavonoids, glycosides, cerebrosides, lignans, and phytosterols have been isolated and identified from different parts of the plant, and these components may produce specific biological effects [11]. A comprehensive review of the phytochemicals in *C. nutans* by Khoo et al. in 2018 reported that (1) the polar extract could have anti-inflammatory, antiviral, anticancer, wound healing, immune, and neuromodulating effects, (2) the semi-polar extract could be promising antiviral, anticancer, and wound healing agents, and (3) the nonpolar extract could have potential anticancer properties [12].

Previous studies on *C. nutans* revealed the presence of β-sitosterol, stigmasterol, and myricyl alcohol. Six known C-glycosyl flavones, namely, vitexin, isovitexin, schaftoside, isomollupentin 7-*O*-β-glucopyranoside, orientin, and isoorientin, have been isolated from the n-butanol and water-soluble portion of the methanolic extract of leaves and stems of *C. nutans* collected in Thailand [13,14]. Likewise, ethanol extract of the leaves yielded C-glycosyl flavones. Among these components, schaftoside, isovitexin, orientin, and vitexin were further identified and quantified, using a two-step thin-layer chromatography and high-performance liquid chromatography protocol. Schaftoside was found to be the major flavone present in samples (concentration ranges from 2.55 mmol/g to 17.43 mmol/g), whereas the other C-glycosyl flavones were present in lower amounts, ranging from isovitexin (0.00–2.01 mmol/g), followed by orientin (0.00–0.86 mmol/g), and vitexin being the lowest amount (0.00–0.91 mmol/g) [15]. As detailed in Section 3 below, these compounds have anti-inflammatory properties when tested in different cells and tissues. Four sulfur-containing compounds, namely clinamides A–C and 2-cis-entadamide A, were also isolated from the ethanol extract from the aerial parts of *C. nutans* [16]. An extraction temperature of 60 °C and an extraction time of 120 min in a water:ethanol solvent ratio of 90:10 *v/v*% resulted in maximal yield of the phenolic compounds and offered the highest anti-radical activity [17]. A mixture of nine cerebrosides and a monoacylmonogalactosylglycerol were also identified in the ethylacetate extract of *C. nutans*. The structures of the cerebrosides were characterized as 1-*O*-β-D-glucosides of phytosphingosines. However, these cerebrosides did not show anti-inflammatory (i.e., anti-cyclooxygenase 1 (COX-1) or anti-cyclooxygenase 2 (COX-2)) activities [13]. Comparison of different extraction techniques, i.e., ethanol, acetone, and chloroform extraction protocols showed that acetone-extracted *C. nutans* contained the highest level of antioxidant potential in terms of its total phenolic and flavonoid activity [18].

In addition to anti-inflammatory properties, *C. nutans* extracts may have anti-cancer effects. A study using triple-negative breast cancer cell line MDA-MB-231 showed no cytotoxic or anti-migratory effect of *C. nutans* ethanolic extracts, but instead, the extract provided significant effects reducing the nuclear factor kB (NF-kB) pathway and expression of proinflammatory cytokines such as interleukin-1β (IL-1β), IL-6, and tumor necrosis factor-α (TNF-α) expression [19].

Direct cytotoxic effects of specific phytochemicals from polar extracts of *C. nutans* have also been found. A novel polysaccharide-peptide complex (CNP-1-2) was obtained from *C. nutans* leaves after hot water extraction followed by ethanol precipitation, and this extract showed a growth inhibitory effect on human gastric cancer cells [20]. One of the fractions of methanol extract of *C. nutans* leaves, identified as triterpenes, has been shown to have anti-proliferative activity on Hep-G2 liver cancer cells [21]. Clinamide D and entadamide C, which were isolated from the powdered bark of the plant via methanol extraction, also showed anticancer effects in two breast cancer cell lines [22].

### 2.2. Non-Polar Extraction

The n-hexane fraction of *C. nutans* was found to have cytotoxic effects in vitro and in vivo. This fraction contained palmitic acid, phytol, hexadecanoic acid, 1-monopalmitin, stigmast-5-ene, pentadecanoic acid, heptadecanoic acid, 1-linolenoylglycerol, and stigmasterol [23]. Three chlorophyll derivatives (phaeophytins) have also been isolated from the chloroform extract of *C. nutans* leaves, which showed inhibitory effect on the herpes simplex virus [24]. Four compounds have been isolated from the hexane fraction of *C. nutans* leaves and identified as schaftoside, stigmasterol, β-sitosterol, and a triterpenoid lupeol. Of these, β-sitosterol was found to have immunosuppressive potential by blocking murine T cell proliferation and secretion of cytokines from T helper 2 cells [25].

### 2.3. Microwave-Assisted and Carbon Dioxide-Assisted Extraction

Phytochemicals have also been extracted from *C. nutans* by a microwave-assisted extraction, pressurized microwave-assisted extraction, supercritical carbon dioxide extraction, and Soxhet method to investigate the best technique for the recovery of phenols, flavonoids, and phytosterols [26]. Results show that microwave-assisted extraction is the best technique to achieve a high yield of polyphenols and flavonoids, whereas supercritical carbon dioxide extraction is the best method for the extraction of phytosterols, including β-sitosterol [26].

## 3. Effects of Individual Phytochemical Components of *C. nutans*

### 3.1. Schaftoside

In the study by De Melo et al., schaftoside (6-C-beta-glucopyranosyl-8-C-alpha-arabinopyranosylapigenin) and vitexin (8-C-beta-glucopyranosylapigenin) isolated from *Eleusine indica* were found to inhibit lung neutrophil recruitment/influx after lipopolysaccharide (LPS)-induced aerosol injury in mice [33]. In another study, schaftoside from *Desmodium styracifolium* was shown to protect mice against acetaminophen-induced hepatotoxicity via regulation of oxidative stress and inflammation. In liver cells, schaftoside was shown to offer both anti-oxidative and anti-inflammatory properties due to its ability to activate the farnesoid X receptor which results in activation of phase II detoxifying enzymes, and regulation of oxidative stress and inflammation [34].

In a study by Zhou et al., it was shown to inhibit mRNA and expressions of pro-inflammatory cytokines IL-1β and IL-6 and TNF-α in microglia induced by subjecting the cells to oxygen glucose deprivation (OGD) [33]. These effects further demonstrated ability of schaftoside to inhibit Toll-like receptor 4 (TLR4) pathway in these cells [35]. Schaftoside was also found to modulate pentylenetetrazol-induced seizure-like behavior in a zebrafish model by suppressing NF-κB and inflammatory cytokine expression [36].

### 3.2. Vitexin

#### 3.2.1. NF-kB and Nrf2 Pathways in Cells and Peripheral Tissues

Intraperitoneal treatment with vitexin was shown to dose-dependently inhibit acetic acid-induced writhing in mice. This could be due to an effect on prevention of decrease of reduced glutathione, ferric-reducing potential, and free-radical scavenger levels [35]. In addition, the sensitivity of vitexin to pain response is linked to its ability to inhibit the production of hyperalgesic cytokines such as IL-1β, IL-6, IL-33, and TNF-α, and upregulate the anti-inflammatory cytokine IL-10 [37]. Vitexin was found to reduce the apoptotic index and the expression of myocardial NF-κB/p65 protein in cardiac muscle cells in a rat model of ischemia/reperfusion injury [38]. Oral administration of vitexin at 30 mg/kg once daily could also inhibit doxorubicin-induced toxicity and increase in inflammatory genes such as NF-κB, IL-1β, IL-6, and TNF-α in the rat heart [39]. Vitexin was reported to protect pancreatic islet cells against LPS-induced inflammation and apoptosis in vivo and in vitro [40]. In a study with rat chondrocytes, vitexin could significantly inhibit the endoplasmic reticulum-activated NF-κB pathway and suppressed the expression of inflammatory cytokines (IL-6 and TNF-α) [41]. It can also modulate the increase in oxidative stress and inflammatory cytokine production after acute LPS-induced lung injury in wild type mice, but not nuclear factor erythroid-2-related factor 2 (Nrf2) knockout mice. These results suggest that the effects of vitexin are dependent on the Nrf2-pathway [42]. Vitexin inhibited the expression of pro-inflammatory mediators IL-1β, IL-6, TNF-α, and matrix metalloproteinases MMP-1, MMP-3, and MMP-13 in chondrocytes derived from osteoarthritis patients. These effects may be due to an effect of vitexin on inhibiting the hypoxia-inducible factor 1α (HIF-1α) signaling pathway [43]. Vitexin decreased the levels of pro-inflammatory cytokines IL-1β and IL-6 and TNF-α in a 4% dextran sodium sulfate-induced model of acute ulcerative colitis [44]. This phytochemical could also reduce LPS-induced liver injury in mice by inhibiting the NF-κB pathway [45]. Vitexin reduced chronic stress and high fat diet-induced hepatic fat deposition and liver inflammation in mice. This could occur via inhibition of the TLR4-NF-κB signaling pathway [46]. Studies on the azoxymethane and dextran sodium sulfate-induced mouse model of ulcerative colitis-associated colorectal cancer showed that oral administration of vitexin significantly improved the clinical signs and symptoms with decreased cytokine production and reduced numbers of macrophages with the M1 pro-inflammatory phenotype in the adjacent non-cancerous tissue [47] These results suggest that the anticancer effect of vitexin in chronic colitis-associated carcinogenesis may be due to its ability to alter the tumor microenvironment [47].

Vitexin was found to inhibit cell-cycle progression in nasopharyngeal carcinoma cells in culture [48]. These effects could be due to an ability of vitexin to inhibit the NF-kB pathway. In this study using a cell-free system, the activity of inhibitor of nuclear factor kappa-B kinase subunit beta (IKKβ) could be suppressed by vitexin, and overexpression of IKKβ could attenuate the inhibitory effect of vitexin [48].

In addition to the NF-kB pathway and production of pro-inflammatory cytokines, vitexin can also interact with the antioxidant pathway involving the nuclear factor erythroid-2-related factor 2 (Nrf2). Here, many phytochemicals can interact the Nrf2/Keap1 complex, and allowing Nrf2 to translocate to the nucleus and interact with the Antioxidant Response Element (ARE). Activation of ARE is responsible for transcription activation of heme oxgenase-1 (HO-1) and other phase II enzymes [49]. In a study by Li et al., vitexin was shown to protect melanocytes from oxidative stress induced by H_2_O_2_ through activating the Nrf2/ARE signaling pathway, and knockdown of Nrf2 reversed the protective effect of vitexin on H_2_O_2_-induced melanocytes [50].

#### 3.2.2. Cells and Vessels in the CNS

Vitexin pretreatment could dose-dependently reduce brain infarct volume in a model of neonatal hypoxia-ischemia, together with an increase in Bcl-2/Bax protein ratio and decrease in the number of TUNEL-positive cells [51]. Vitexin could inhibit oxidative damage in Neuro-2a neurons exposed to glutamate excitotoxicity by augmenting the Nrf2/heme oxygenase-1 (HO-1) pathway, upregulation of glutamate transporters, and downregulation of calpain and NMDA receptors [52]. Vitexin could also offer protective effects on Neuro-2a cells treated with the cytotoxic Aβ25-35, by suppressing ROS-mediated lipid peroxidation and modulating the expression of genes involved in antioxidant response mechanisms (Nrf2, HO-1), cholesterol metabolism (LXR-α, APOE, ABCA-1, Seladin-1), and endoplasmic reticulum stress (Grp78, Gadd153) [53]. Vitexin reduced cellular injury and increased the expression of tight junction protein ZO-1 in rat brain microvascular endothelial cells after oxygen-glucose deprivation [54]. These effects could be attributed to alteration of the Na^+^-K^+^-Cl^−^ co-transporter 1 and F actin expression. Moreover, the ability of vitexin to suppress excessive brain electrical activity in the neonatal rat brain after carotid artery occlusion led to the suggestion that this compound could be a potential therapeutic for treatment of neonatal epilepsy [54]. In a study on ischemic stroke in rats using the middle cerebral artery occlusion (MCAO) model, vitexin treatment reduced brain infarct volume and decreased oxidative damage and pro-inflammatory cytokine IL-6 and TNF-α secretion [54]. Vitexin could also inhibit MCAO-induced decreased expression of peroxisome proliferator-activated receptor γ (PPARγ) and p62 [55]. In a study with zebrafish embryos treated with acrylamide, a pro-inflammatory agent, vitexin could reduce behavioral changes and modulate the release of pro-inflammatory mediators [56]. Vitexin also reduced septicemia-induced downregulation of chemokines CXCL1 and CX3CL1, and expression of pro-inflammatory mediators such as NF-κB p65, MCP-1, IL-6, IL-8. and TNF-α in endothelial cells [57]. These effects could reduce neutrophil recruitment and inflammation during septic encephalopathy [57]. Vitexin could suppress inflammatory changes and endothelial damage in mouse carotid arteries subjected to disturbed blood flow [58]. These results were thought to occur via an interaction between vitexin and apurinic/apyrimidinic endonuclease 1 that prevents the acetylation and translocation of the endonuclease to the nucleus and subsequently initiating inflammation via the NF-kB pathway [58]. In mice fed with a high-fat diet, vitexin was shown to upregulate antioxidant enzymes such as superoxide dismutase and catalase and decrease the levels of malondialdehyde, an oxidative stress marker, as well as expression of pro-inflammatory mediators IL-1β and TNF-α in the brain [59]. Furthermore, there is evidence that vitexin could modulate cognitive defects and suppress neuronal injury after chronic cerebral hypoperfusion in rats [60]. It could also reduce NLRP3-mediated inflammation and improve cell viability in HT22 mouse hippocampal neurons subjected to oxygen-glucose deprivation (OGD) [60].

### 3.3. Isovitexin

#### 3.3.1. Peripheral Organs

Isovitexin has antioxidant activity that is reported to be on par with that of α-tocopherol. This phytochemical was found to reduce TNF-α, prostaglandin E2 (PGE2), and COX-2 expression in LPS-activated RAW 264.7 macrophages [61]. Isovitexin also attenuated histopathological changes, infiltration of polymorphonuclear granulocytes, and endothelial cell activation in an LPS-induced model of acute lung injury. Isovitexin could inhibit MAPK phosphorylation, reduce NF-κB nuclear translocation, and upregulate Nrf2 and HO-1 expression in RAW 264.7 cells [62,63]. The phytochemical also protected against LPS/D-Gal-induced liver injury by inhibiting oxidative stress and inflammatory responses [64], and decreased cisplatin-induced kidney injury [65]. These effects are thought to occur via a decrease in the NF-κB pathway and an increase in the Nrf2-HO-1 pathway [65]. Isovitexin also inhibited TNF-α expression in LPS-incubated RAW 264.7 monocyte/macrophage-like cells [66].

#### 3.3.2. Blood Vessels and the CNS

Isovitexin was found to suppress TNF-α induced expression of ICAM-1 and VCAM-1 in human umbilical vein endothelial cells [67]. This phytochemical could also reduce the expression of M1 pro-inflammatory markers from BV-2 microglia and increase the expression of M2 anti-inflammatory markers such as IL-10 from these cells. These effects were thought to be associated with the ability for vitexin to induce PPAR-γ activation and the CaMKKβ/AMPK-PGC-1α pathway [68].

### 3.4. Orientin

#### 3.4.1. Peripheral Organs

Orientin was shown to reduce LPS-induced upregulation of pro-inflammatory markers including IL-1β, IL-6, IL-18, and TNF-α and increase in prostaglandin E2 (PGE2) and nitric oxide (NO), in RAW 264.7 macrophage cells. This phytochemical could also reduce the expression of COX-2 and inducible nitric oxide synthase (iNOS) as well as the NF-κB pathway and nucleotide-binding domain-like receptor protein 3 (NLRP3) inflammasome activation [69]. Orientin was also shown to alleviate hydrogen peroxide-stimulated cytotoxicity, inhibit reactive oxygen species (ROS) generation, and prevent glutathione depletion in RAW 264.7 cells. These effects were mostly abolished after siRNA knockdown of Nrf2 and HO-1, suggesting the involvement of the Nrf2-HO-1 signaling pathway [70]. Orientin could reduce inflammation by inhibiting mast cell degranulation and release of histamine and pro-inflammatory cytokines [71]. It was found to protect against acute lung injury by suppressing inflammation and oxidative stress. These effects could occur via suppression of the NF-kB pathway and activation of the Nrf2-HO-1 pathway [72]. Orientin also downregulated the expression of inflammatory genes, IL-6, TNF-α, NF-kB, and COX-2, as well as iNOS in the 1,2-dimethyl hydrazine-induced colorectal cancer model in rats [73].

#### 3.4.2. Blood Vessels and the CNS

Orientin and isoorientin showed vascular barrier protective effects by reducing the shedding of endothelial cell protein C receptor from the cell surface of endothelial cells [74]. Orientin was also found to inhibit high glucose-induced vascular inflammation in vitro and in vivo, suggesting that it may have beneficial effects in the treatment of diabetic vascular complications [75]. Orientin-2″-*O*-galactopyranoside significantly reduced the production of NO and TNF-α in LPS-stimulated BV-2 microglial cells. This compound could also inhibit LPS-induced expression of NF-kB, IL-1β, COX-2, and iNOS, and upregulate the Nrf2/HO-1 antioxidant pathway in these cells [76]. Part of the anti-inflammatory effect of orientin on blood vessels may be due to its ability to inhibit high mobility group box-1 protein. The latter could induce cytoskeletal rearrangements in endothelial cells and result in increased vessel permeability during systemic inflammation [77]. Orientin could inhibit the expression and/or activity of two of the major isoforms of phospholipase A_2_, i.e., cytosolic phospholipase A_2_ (cPLA_2_) and secretory phospholipase A_2_ (sPLA_2_) in LPS-treated human umbilical vein endothelial cells [78]. Intraperitoneal injection of orientin (5 mg/kg) was found to ameliorate cognitive deficits in mice induced with the Alzheimer’s disease peptide, Aβ1-42. These effects were associated with nuclear translocation of Nrf2, enhanced expression of HO-1, attenuation of mitochondrial dysfunction, and reduced oxidative stress markers such as 4-hydroxynonenal (4-HNE) and 8-hydroxy-2’-deoxyguanosine (8-OHdG) [79]. Orientin was also reported to improve neuron morphology and decrease expression of aquaporin-4 and inflammatory molecules as well as infarct volume in the brain, after cerebral ischemia-reperfusion injury in rats [80]. The phytochemical was found to alleviate thermal and mechanical allodynia in rats after spinal nerve ligation, a condition correlated with modulation of microglia and astrocyte activation, and inhibition of the TLR4/NF-κB signaling pathway [81]. Orientin could also inhibit oxidized LDL-induced increases in NF-κB, IL-1β, IL-6, and TNF-α expression in RAW 264.7 macrophages. This effect may be partly due to downregulation of the oxidized low-density lipoprotein receptor CD36 [82]. Orientin and isoorientin were found to produce strong inhibition (≥70%), while isovitexin and vitexin moderate inhibition (50–69%) on IL-1β, IL-6, COX-2, and PGE2 expression/activities at 25 and 50 μM concentrations [83].

### 3.5. Isoorientin

#### 3.5.1. Peripheral Organs

Isoorientin has significant anti-inflammatory properties at a dose of 30 mg/kg in the carrageenan-induced hind paw edema model without inducing any apparent acute toxicity [84]. Isoorientin obtained from the leaves of *Sasa borealis* was found to upregulate and activate Nrf2 and offer protective effects against reactive oxygen intermediates in HepG2 human liver cancer cell line. Isoorientin could also induce increases in levels of antioxidant enzyme proteins. The cytoprotective and antioxidative effects of isoorientin appear to involve the PI3K/Akt pathway [85]. Isovitexin could alter lipid metabolism and reduce oxidative stress and inflammatory mediators such as IL-1, IL-6, and TNF-α in the liver of high-fructose treated hyperlipidemic mice [86]. The phytochemical also reduced the expression of IL-1β, IL-6, and TNF-α via inhibiting NF-κB activation after carbon tetrachloride-induced liver injury in rats [87]. Isoorientin was found to reduce tissue edema in the mouse carrageenan injection model of inflammation. This effect could be due to its ability to decrease the levels of pro-inflammatory mediators such as IL-1β, IL-6, and TNF-α and COX-2, and increase levels of antioxidant enzymes, including catalase and glutathione S-transferase [88]. The phytochemical was also found to upregulate the expression of sirtuin 1 and sirtuin 6 in vivo and in vitro and had a protective effect on cisplatin-induced nephrotoxicity. These effects of isoorientin were abolished in Nrf2 knockout mice [89]. JAK2/STAT3 pathway inhibitors were also shown to inhibit the anti-inflammatory effect of isoorientin in a model of sepsis-induced acute lung injury [90].

#### 3.5.2. Blood Vessels and the CNS

**Figure 2:**
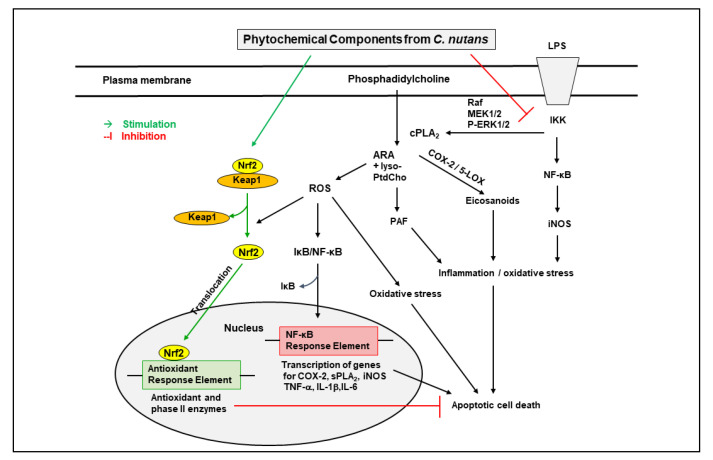
Inflammatory and oxidative stress pathways targeted by *C. nutans* and its phytochemical components. Abbreviations: LPS: lipopolysaccharide; IKK: inhibitor of kappaB kinase; Raf: rapidly activated sarcoma; MEK1/2: mitogen activated protein kinase 1/2; P-ERK1/2: phosphorylated extracellular signal related kinase 1/2; NF-kB: nuclear factor kappa-light-chain-enhancer of activated B cells; iNOS: inducible nitric oxide synthase; cPLA_2_: cytosolic phospholipase A_2_; COX-2: cyclooxygenase 2; 5-LOX: 5-lipoxygenase; ARA: arachidonic acid; lyso-PtdCho: lysophosphatidylcholine; PAF: platelet activating factor; sPLA_2_: secretory phopholipase A_2_, TNF-α: tumor necrosis factor-alpha; IL-1β: interleukin-1β; Nrf-2: nuclear factor erythroid 2–related factor 2; KEAP1: Kelch-like ECH associated protein 1.

Isoorientin could attenuate LPS-induced pro-inflammatory responses including expression of iNOS, COX-2, and pro-inflammatory cytokines such as TNF-α and IL-1β, through down-regulation of ROS-related MAPK/NF-κB signaling pathway in BV-2 microglial cells [91]. Treatment with isoorientin (5 and 10 mg/kg, p.o.) significantly improved cognitive impairment, and decreased acetylcholinesterase and thiobarbituric acid reactive substance activities in the hippocampus and frontal cortex of scopolamine injected mice. These results suggest an antioxidant and memory-enhancing effect of isoorientin [92]. Isoorientin increased the expression of tight junction proteins occludin and ZO-1, in the brain of endotoxemic mice. The phytochemical also decreased activation of ERK and NF-κB and expression of COX-2, and increased activation of Nrf2/HO-1, in LPS-induced RAW264.7 cells. These results suggest an effect of isoorientin in reducing inflammation and protecting the integrity of the blood–brain barrier in endotoxemic mice [93]. Isoorientin could also modulate Aβ25-35-induced increase in pro-inflammatory mediators such as IL-6, TNF-α, and COX-2, as well as ROS production in BV-2 microglial cells [94]. The inflammatory and oxidative stress pathways targeted by *C. nutans* extracts and their phytochemical components are indicated in Figure 2.

## 4. *Clinacanthus nutans* Extracts on Inflammatory Pathways in Cerebral Ischemia and Neurovascular Systems

### 4.1. Clinacanthus nutans on Cerebral Ischemia

*Clinacanthus nutans* extracts have been shown to affect the expression and activity of cPLA_2_, an important enzyme in inflammation. This enzyme is known for hydrolysis of glycerophospholipids to produce arachidonic acid and lysophospholipids, and in turn, metabolism of arachidonic acid produces pro-inflammatory mediators and eicosanoids, as well generation of free radicals [95,96,97]. The expression cPLA_2_ mRNA is regulated by histone acetylation. In a study by Tan et al., *C. nutans* extract as well as NU9056, a Tip60 histone acetylase (HAT) inhibitor, could suppress cPLA_2_ mRNA expression in neurons due to OGD [98]. *C. nutans* extracts also suppressed histone deacetylase 1 (HDAC1) and HDAC6 transcription and ameliorated neuronal death and astrocyte/endothelial damage due to OGD [99]. In another study, *C. nutans* extract was found to protect neurons and ameliorate ischemic injury by promoting the anti-apoptotic activity of peroxisome proliferator-activated receptor-gamma (PPAR-γ), a stress-induced transcription factor. The augmentation of transcription by *C. nutans* extract appeared to occur through selective increase of CCAAT/enhancer binding protein (C/EBP) β binding to a specific C/EBP binding site on the PPAR-γ promoter [100]. PPAR-γ stimulation is known to downregulate the NF-kB pathway, thus inhibiting the expression of a wide array of pro-inflammatory mediators in microglia and macrophages, including IL-1β, IL-6, IL-12, TNF-α, COX-2, and iNOS [101,102]. Transient OGD led to induction of IL-1β, IL-6, and TNFα, and pretreatment with *C. nutans* extract suppressed the production of these inflammatory cytokines in primary neurons. *C. nutans* extract also inhibited IL-1β transcription through NF-κB/p65 nuclear translocation, and siRNA knockdown of either p65 or IL-1β mitigated OGD-mediated neuronal death. Post-ischemic treatment of *C. nutans* attenuated IκBα degradation and decreased IL-1β, IL-6, and TNFα production in the ischemic brain [103].

### 4.2. Clinacanthus nutans on Neurovascular Systems

*Clinacanthus nutans* extracts could also show potential effects against vascular dementia. Oral administration of *C. nutans* leaf water extract (500 and 1000 mg/kg) was found to reduce sickness behavior and brain inflammatory markers in rats after intracerebral injection of LPS [104]. *Clinacanthus nutans* ethanolic extract was found to have anti-apoptotic effects against LPS-induced damage in bovine endothelial cells. This could be due to an effect of its components, glyceryl 1,3-disterate, kaempferol 3-*O*-feruloyl-sophoroside 7-*O*-glucoside, and hydroxypthioceranic acid, which showed pro-surviving Bcl-2 and pro-apoptotic Bax expression activities [30]. *Clinacanthus nutans* aqueous extracts could reduce LPS-induced increase in proinflammatory IL-1β expression but increase anti-inflammatory IL-2 and IL-4 expression in brain tissue after oral administration in rats [105]. A recent study showed that *C. nutans* leaf, but not stem, extracts had a significant effect in reducing 7-kecoholesterol-induced brain endothelial cell death and suppressed inflammatory responses in endothelial cells, including reducing IL-1β, IL-6, IL-8, TNF-α, and COX-2 expression [106]. Together, the above results suggest a protective effect of *C. nutans* in neurovascular injury by reducing the expression of pro-inflammatory enzymes such as cPLA_2_ and increasing the expression of cytoprotective enzymes.

## 5. Conclusions and Future Directions

Studies with polar and nonpolar extraction procedures for leaves of *C. nutans* indicated different extracts yielded components with anti-inflammatory, anti-tumorigenic, and antiviral properties. There are increasing studies linking these extracts against disorders in cells and tissues in the central and peripheral systems. Future studies to standardize the extraction protocols and to associate them with treatment of specific disorders will help to direct better use of the products for health and diseases.

Analyses of extracts from *C. nutans* leaves have led to identification of C-glycosyl flavones including schaftoside, isovitexin, orientin, and vitexin. A review of studies on individual phytochemicals indicated anti-inflammatory and antioxidative properties to different extents. In general, anti-inflammatory properties are linked to their ability to suppress the NF-kB pathway and production of iNOS as well as pro-inflammatory cytokines, such as IL-1β and TNF-α. In addition, anti-inflammatory properties are also linked to ability to suppress the cPLA_2_ pathway associated with increase of COX-2 and pro-inflammatory eicosanoids. To some extent, individual phytochemicals also exhibit antioxidative properties as demonstrated by their ability to upregulate the antioxidative stress pathway involving Nrf2 and production of HO-1. The ability for the *C. nutans* extracts and their components to modulate these pathways under different inflammatory conditions has not been quantitatively defined. Therefore, more studies are needed to understand the molecular mechanism of action. Development of reporter gene assays targeting the transcription factors will be useful to better define properties of the pathways and aid development of therapeutic potential for treatment of diseases.

## Figures and Tables

**Table 1:** Solvents used in extraction and phytochemicals obtained from *C. nutans*.

Solvent	Phytochemical Components	Potential Health Effects	References
Distilled water	Glucoside Sulfur-containing compounds Phytosterols Triterpenoids Flavones Amino acids	Anti-inflammatory	[27]
Methanol	Schaftoside Isoorientin Orientin Isovitexin Vitexin	Anti-inflammatory	[6]
Methanol	Entadamide C Clinamide D	Anticancer	[22]
Methanol	Palmitic acid Phytol 1-Monopalmitin Stigmast-5-ene Pentadecanoic acid Heptadecanoic acid 1-linolenoylglycerol Glycerol monostearate Alpha-tocospiro B Stigmasterol	Anti-diabetic	[28]
Methanol	Betulin Stigmasterol Sitosterol β-Amyrin Vitamin E Campesterol	Anticancer	[29]
Methanol	Schaftoside Isomollupentin 7-*O*-β-glucopyranoside Orientin Isoorientin Vitexin Isovitexin		[14]
Ethanol	Glyceryl 1,3-disterate ester (C_39_H_76_O_5_), Kaempferol 3-*O*-feruloyl-sophoroside 7-*O*-glucoside (C_43_H_48_O_24_) Hydroxypthioceranic acid (C_46_H_92_O_3_)	Antiapoptotic	[30]
Ethanol	Myricetin Orientin Isoorientin Vitexin Isovitexin Apigenin Ferulic acid		[10]
Ethanol	Clinamides 2-*cis*-entadamide		[16]
Ethanol	Schaftoside Orientin Isovitexin Vitexin	Anti-inflammatory	[15]
Chloroform	Purpurin-18 phytyl ester	Anti-inflammatory	[31]
Dichloromethane	Palmitic acid Linolenyl alcohol	Anticancer	[32]
Hexane	Palmitic acid Phytol Hexadecanoic acid 1-Monopalmitin Stigmast-5-ene Pentadecanoic acid Heptadecanoic acid 1-Linolenoylglycerol Stigmasterol		[23]
Hexane	Schaftoside Stigmasterol β-sitosterol Triterpenoid lupeol	Immuno-modulatory	[25]
Hexane	13(2)-hydroxy-(13(2)-R)-phaeophytin b, 13(2)-hydroxy-(13(2)-S)-phaeophytin a 13(2)-hydroxy-(13(2)-R)-phaeophytin a.	Antiviral	[24]
Ethyl acetate	Lupeol Lup-20(29)-en-3-one Lup-20(29)-en-ol acetate Stigmasterol Sitosterol Betulin Campesterol Squalene Vitamin E Oleic acid	Anticancer	[29]
Microwave Assisted Extraction	Polyphenols Flavonoids		[26]
Supercritical carbon dioxide	Phytosterols β-Sitosterol		[26]
